# Blood Lead Concentration and Thyroid Function during Pregnancy: Results from the Yugoslavia Prospective Study of Environmental Lead Exposure

**DOI:** 10.1289/ehp.1307669

**Published:** 2014-05-27

**Authors:** Linda G. Kahn, Xinhua Liu, Biljana Rajovic, Dusan Popovac, Sharon Oberfield, Joseph H. Graziano, Pam Factor-Litvak

**Affiliations:** 1 Department of Epidemiology, and; 2 Department of Biostatistics, Mailman School of Public Health, Columbia University, New York, New York, USA; 3 Regional Hospital Kosovska Mitrovica, Mitrovica, Kosovo; 4 Department of Medicine, University of Pristina, Pristina, Kosovo; 5 Department of Pediatrics, College of Physicians and Surgeons, Columbia University, New York, New York, USA; 6 Department of Environmental Health Sciences, Mailman School of Public Health, Columbia University, New York, New York, USA

## Abstract

Background: Although maternal hypothyroidism increases the risk of adverse neonatal and obstetric outcomes as well as lower IQ in children, the environmental determinants of maternal thyroid dysfunction have yet to be fully explored.

Objectives: We aimed to examine associations between mid-pregnancy blood lead (BPb) and concomitant measures of thyroid function among participants in the Yugoslavia Prospective Study of Environmental Lead Exposure.

Methods: As part of a population-based prospective study of two towns in Kosovo—one with high levels of environmental lead and one with low—women were recruited during the second trimester of pregnancy, at which time blood samples and questionnaire data were collected. We measured concentrations of BPb, free thyroxine (FT_4_), thyroid-stimulating hormone (TSH), and thyroid peroxidase antibodies (TPOAb) in archived serum samples.

Results: Compared with women from the unexposed town, women from the exposed town had lower mean FT_4_ (0.91 ± 0.17 vs. 1.03 ± 0.16 ng/dL), higher mean TPOAb (15.45 ± 33.08 vs. 5.12 ± 6.38 IU/mL), and higher mean BPb (20.00 ± 6.99 vs. 5.57 ± 2.01 μg/dL). No differences in TSH levels were found. After adjustment for potential confounders, for each natural log unit increase in BPb, FT_4_ decreased by 0.074 ng/dL (95% CI: –0.10, –0.046 ng/dL), and the odds ratio for testing positive to TPOAb was 2.41 (95% CI: 1.53, 3.82). We found no association between BPb and TSH.

Conclusions: Prolonged lead exposure may contribute to maternal thyroid dysfunction by stimulating autoimmunity to the thyroid gland.

Citation: Kahn LG, Liu X, Rajovic B, Popovac D, Oberfield S, Graziano JH, Factor-Litvak P. 2014. Blood lead concentration and thyroid function during pregnancy: results from the Yugoslavia Prospective Study of Environmental Lead Exposure. Environ Health Perspect 122:1134–1140; http://dx.doi.org/10.1289/ehp.1307669

## Introduction

The adverse effects of early childhood exposure to high levels of environmental lead are well established (Needleman and Landrigan 1991). In some but not all studies, higher prenatal lead exposure [blood lead (BPb) level, 10–20 μg/dL] is associated with a wide range of adverse pregnancy outcomes (Bellinger 2005), including shorter gestational lengths (Cantonwine et al. 2010); reduced birth weight (Bellinger et al. 1991; Gonzalez-Cossio et al. 1997), birth length, and head circumference (Hernandez-Avila et al. 2002); deficits in infant mental development (Gomaa et al. 2002); and decreased child IQ (Schnaas et al. 2006; Wasserman et al. 1998, 2000). Elevated prenatal exposure to lead may be associated with adult-onset psychiatric disorders such as schizophrenia (Opler et al. 2004, 2008). Although mean BPb levels in the United States declined precipitously following the removal of lead from gasoline and most paint in the mid-1970s, the greatest decline in IQ among children occurs at the lowest levels of exposure (Lanphear et al. 2005), indicating that there may be no safe level of lead exposure (Bellinger 2008). In large areas of the world, where the mining, smelting, and refining of lead and the manufacture and recycling of lead-containing products such as batteries, computers, and solar panels are not closely monitored, lead poisoning is still a serious health concern for children. A recent episode of acute lead poisoning related to artisanal gold processing in a village in northwestern Nigeria that killed 25% of the population < 5 years of age emphasizes the hazard that lead continues to pose in many places around the world (Dooyema et al. 2012).

A recent report on a U.S. national sample of more than half a million pregnant women found that 15.5% of those screened tested positive for either clinical [elevated thyroid-stimulating hormone (TSH) and reduced free thyroxine (FT_4_)] or subclinical (elevated TSH and normal FT_4_) hypothyroidism, far higher than previous estimates (Blatt et al. 2012). Prevalences in other parts of the world, especially developing countries where iodine deficiency is still a public health problem, have been found to be even greater (Mbah et al. 2011). Despite its high prevalence and negative outcomes, little is known about the predictors of clinical and subclinical gestational hypothyroidism aside from iodine deficiency. Maternal iodine intake must increase by 50% to fuel the increase in thyroid hormone production during pregnancy (Stagnaro-Green and Pearce 2013), and even mild to moderate first-trimester gestational iodine deficiency can lead to decrements in verbal IQ and reading ability in school-age children (Bath et al. 2013). Other variables reported to be associated with gestational hypothyroidism include larger maternal thyroid size, higher gravidity, higher prepregnancy body mass index (BMI), and increased fetal gestational age (Boas et al. 2009a; Mbah et al. 2011). Animal studies and studies of acute human exposure indicate that numerous chemicals interfere with thyroid hormone regulation and function (Boas et al. 2009b; Hartoft-Nielsen et al. 2011; Pearce and Braverman 2009). However, few studies assess the associations between persistent lower-dose environmental exposures on thyroid function, and even fewer consider these in pregnant women.

The deleterious effect of gestational hypothyroidism on fetal brain development is well documented (de Escobar et al. 2004). Additionally, the presence of maternal thyroid peroxidase antibodies (TPOAb) during late gestation has been associated with reduced child IQ at 5 years of age even when controlling for postpartum thyroid dysfunction and maternal depression (Pop et al. 1995). Although untreated maternal thyroid dysfunction has been associated with a reduction of up to seven IQ points in school-age children (Haddow et al. 1999), results of studies in which mothers were treated have been inconsistent. Man and Serunian (1976) found lower psychological scores among 7-year-old children of mothers with inadequately treated prenatal hypothyroxinemia compared with the children of adequately treated and euthyroid women; and Lazarus et al. (2012) recently reported comparable mean IQ scores at 3 years of age among children whose mothers were randomized to be screened and, if necessary, treated for gestational hypothyroidism compared with children whose mothers were not screened or treated.

Building on previous occupational studies and on studies in small general population samples (Bledsoe et al. 2011; Dundar et al. 2006; Mbah et al. 2011; Tuppurainen et al. 1988), we hypothesized that maternal BPb might be associated with reduced thyroid function via one of three possible pathways. One potential mechanism involves iodine, adequate levels of which are essential for normal thyroid function. More than half a century ago, Slingerland (1955) demonstrated impaired uptake of iodine by fresh sheep thyroid tissue exposed to lead nitrate in solution. A subsequent study by Sandstead et al. (1969) of individuals exposed to lead either occupationally or through ingestion of tainted whiskey demonstrated a similar association in humans. The second pathway involves disruption of the release of transthyretin (TTR) into the cerebrospinal fluid, preventing the transport of FT_4_ to the brain. Lead is sequestered in the choroid plexus, the region of the brain where brain-specific TTR is produced. In both rodent and human studies, Zheng et al. (1996, 2001) have shown BPb to be inversely associated with both TTR and FT_4_. In both of these scenarios, we would expect to see elevated maternal mid-pregnancy TSH, reduced mid-pregnancy FT_4_, and no discernible TPOAb. Finally, we speculated that lead may affect thyroid function by triggering autoimmune thyroiditis (AT). In the case of such a direct assault on the thyroid gland, we would expect to see elevated TPOAb and depressed FT_4_, but no effect on TSH.

To test these three potential pathways, we examined the associations between BPb and measures of FT_4_, TSH, and TPOAb in data collected during the Yugoslavia Prospective Study of Lead Exposure, Pregnancy Outcomes, and Child Development (Graziano et al. 1990). To our knowledge, this is the first study to explore the relationship between lead exposure and thyroid function in a sample of pregnant women.

## Methods

**Figure 1:**
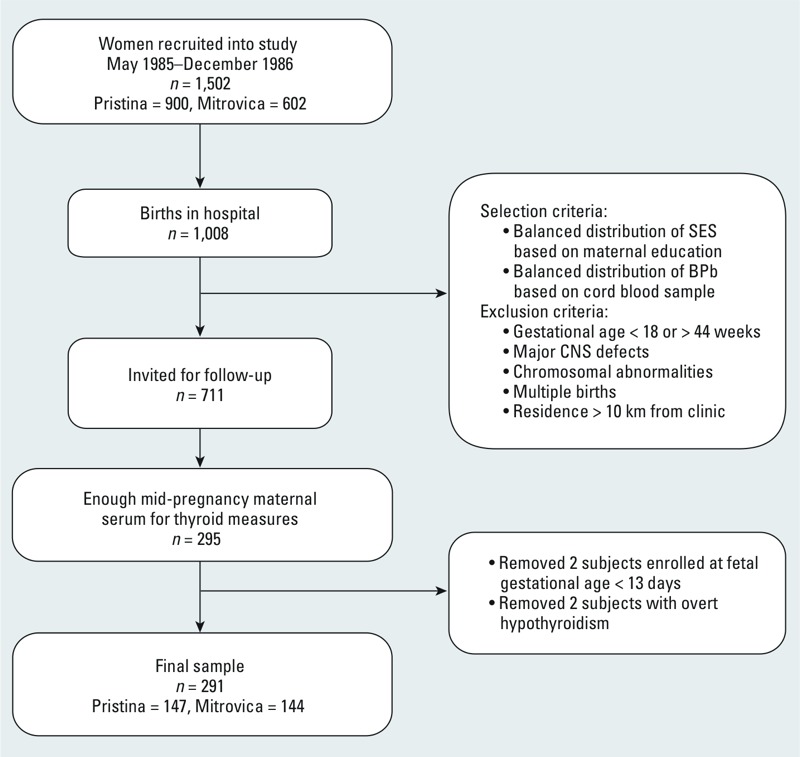
Recruitment and participation of study subjects. Abbreviations: CNS, central nervous system; SES, socioeconomic status.

*Study population*. Between May 1985 and December 1986, women in their second trimester of pregnancy were invited to participate in a study of pregnancy outcomes at their first prenatal visit to government clinics located at the centers of two towns in Kosovo. Details of the study design have been published previously (Factor-Litvak et al. 1991, 1999; Graziano et al. 1990; Wasserman et al. 1998). A total of 1,502 women were recruited: 602 from Mitrovica, a town with a lead smelter, refinery, and battery plant in which high BPb concentrations had been reported in both adults and children (Popovac et al. 1982); and 900 from Pristina, 25 miles to the south, where the primary source of environmental lead was gasoline (lead-based paint has been banned in Yugoslavia since 1922). Complete delivery data were available on 1,008 mother–infant pairs. Inclusion criteria for continuing in the study were giving birth to a single child between 18 and 44 weeks gestation who was free of major central nervous system defects or chromosomal abnormalities, and living within 10 km of the clinic. The 394 infants from Mitrovica with available cord blood BPb measures were then divided into three groups: < 15 μg/dL, 15–20 μg/dL, and > 20 μg/dL. Two groups of infants from Pristina were selected for follow-up: one group frequency-matched on BPb concentration to the group from Mitrovica with BPb < 15 μg/dL, and a second group matched on maternal and paternal education to the group from Mitrovica with BPb > 20 μg/dL. Of the resulting 711 infants invited to continue in the study, the parents of 541 consented. The sample for the present analyses included 291 women enrolled in mid-pregnancy who had adequate serum in storage to measure FT_4_, TSH, and TPOAb levels at the time of a follow-up study of prenatal thyroid function, lead, and child growth at 7 years of age (Lamb et al. 2008), and did not display overt hypothyroidism, defined as TSH > 2.5 μIU/mL and FT_4_ < 0.7 ng/dL, the latter cut-off value representing the lowest 5th percentile of the sample population (Stagnaro-Green et al. 2011) (Figure 1). This study was approved by the Columbia University Institutional Review Board (IRB) and by the “Komitetietik,” a National Institutes of Health–registered IRB at the University of Pristina, Kosovo. All women gave written informed consent before the study.

*Data collection*. At their first prenatal visit, pregnant women enrolled in this study were interviewed by trained bilingual (Serbo-Croatian and Albanian) interviewers who collected data on sociodemographic criteria, pregnancy and health history, and lifestyle variables. Fetal gestational age at interview ranged from 9 to 28 weeks, with a mean of 18 completed weeks. The nurses measured the women’s height and weight and obtained venous blood samples, which were refrigerated on site and transported on wet ice to Columbia University. After transport to Columbia, blood was stored at –20°C for several months until analyzed for lead, hemoglobin, erythrocyte protoporphyrin, and serum ferritin; samples with evidence of hemolysis were excluded. The remaining blood and serum was stored at –20°C and the thyroid measures were analyzed approximately 15 years after collection. Pilot data indicated that the values of FT_4_, TSH, and TPOAb were in the range expected for women during mid-pregnancy.

Blood lead. Mid-pregnancy maternal serum samples were assayed for BPb according to methods described previously (Factor-Litvak et al. 1991). The Columbia laboratory participates in the Centers for Disease Control and Prevention (CDC) quality control program for BPb analyses and is certified by the Occupational Safety and Health Administration; during the course of the study, the intraclass correlation coefficient for agreement with CDC values for BPb was 0.95. All samples had BPb levels above the detection limit of 0.1 μg/dL.

Maternal thyroid measures. Maternal thyroid function during pregnancy was assessed using FT_4_, TSH, and TPOAb, all of which have been shown to resist deterioration during freezing, storage, and thawing (Mannisto et al. 2007). FT_4_ and TPOAb were measured by a radioimmunoassay procedure, and TSH was measured using an IRMA procedure (all from ICN Biomedicals, Costa Mesa, CA). According to the technical specifications of the assay, TPOAb was characterized as slightly elevated if TPOAb levels were ≥ 10 IU/mL and < 20 IU/mL, moderately elevated if ≥ 20 IU/mL and < 100 IU/mL, and highly elevated if ≥ 100 IU/mL. For this study, all cases with slightly, moderate, or highly elevated TPOAb levels were considered positive. Euthyroid women with elevated TPOAb levels were considered at risk of hypothyroidism (Stagnaro-Green et al. 2011).

*Statistical analyses*. We natural log (ln)–transformed BPb, TSH, and TPOAb to meet assumptions of the statistical models and to reduce the influence of extreme values. Preliminary analyses evaluated potential confounding variables including maternal age, fetal gestational age at blood sample [because measures of thyroid hormone vary during the course of pregnancy (Glinoer 2000)], town (to account for unspecified geographic factors that might influence thyroid hormone level in pregnancy), anthropometric measures (maternal height, prepregnancy weight, and BMI), hemoglobin (Hgb), lifestyle characteristics (smoking, alcohol use, and coffee consumption), and sociodemographics (ethnicity, maternal education, parity, ratio of rooms to number of adults in household, and home ownership). Specifically, we used analysis of variance (ANOVA) to compare means of continuous outcome variables according to levels of categorical predictor variables. We calculated Spearman correlation coefficients to assess bivariate associations between continuous predictor and outcome variables. Multiple linear regression analysis was used to estimate covariate-adjusted associations between BPb and the continuous outcome measures of thyroid function, and logistic regression analysis to assess the relationship between BPb and the binary outcome measure TPOAb. Outcome-specific covariates were identified in preliminary analyses as variables associated with BPb and the specific outcome at *p* < 0.2. We also identified as covariates those found in previous studies to be associated with the outcome (Boas et al. 2009a; Mbah et al. 2011). We graphically examined the relationships between BPb and outcome measures and additionally ran our regression models substituting town for BPb as the main predictor variable. In sensitivity analyses restricted to Albanian women, associations between BPb and thyroid outcome measures were unchanged, indicating that ethnicity was not a major confounder (data not shown). We also found no difference when we included a quadratic term for fetal gestational age in our models and concluded that our results were not affected by a nonlinear association between gestational age and thyroid measures (data not shown). All statistical tests were two-tailed, with a significance level of 0.05. Data were analyzed using SAS® 9.2 statistical software (SAS Institute Inc., Cary, NC).

## Results

At recruitment, the 291 subjects used in this analysis were similar to the 420 members of the cohort who did not meet the inclusion criteria in terms of age, education, number of prior live births, mid-pregnancy BPb and Hgb levels, and fetal gestation age at mid-pregnancy blood draw. Women from the two towns were comparable on all of these measures except for BPb levels. The only notable difference between those included and not included is that the distribution of ethnicities between the two towns, which had been comparable at the time of recruitment, was no longer comparable after loss to follow-up over the subsequent 7 years, likely due to migration during the mounting ethnic tensions in the late 1980s and early 1990s. In Pristina, the proportion of Albanian participants increased (from 59.0% in the original sample to 70.8% after loss to follow-up), the proportion of Serbian participants decreased (from 28.5% to 22.5%), and the proportion of other ethnicities decreased (from 12.5% to 6.8%), whereas in Mitrovica, the distribution did not change (53.4% vs. 54.9% Albanian, 28.65% vs. 27.1% Serbian, 18.0% vs. 18.1% other). In Mitrovica, those included had slightly higher mid-pregnancy BPb compared with those lost to follow-up (20.0 vs. 18.5 μg/dL), and among those included in the study, women in Mitrovica had slightly fewer prior live births compared with those in Pristina (mean, 1.4 vs. 1.7), but neither of these differences reached statistical significance (Table 1).

**Table 1:** Participants compared with members of the Yugoslavia Prospective Study of Environmental Lead Exposure cohort lost to follow-up by child age 7 years.

Characteristic	Included				Lost to follow-up			
	Pristina (*n* = 147)	Mitrovica (*n* = 144)	Range	*p*-Value	Pristina (*n* = 165)	Mitrovica (*n* = 255)	Range	*p*-Value
Maternal age (years)	26.6 ± 4.7	26.7 ± 5.2	16.1–41.7	0.87	26.7 ± 4.5	26.1 ± 4.7	15.1–46.0	0.19
Maternal education (years)	9.2 ± 3.9	9.3 ± 3.8	0–17	0.87	9.8 ± 3.8	9.4 ± 4.0^*a*^	0–17	0.29
No. of prior live births	1.7 ± 1.7	1.4 ± 1.6	0–9	0.099	1.5 ± 1.4	1.4 ± 1.5	0–9	0.53
Mid-pregnancy BPb (μg/dL)	5.6 ± 2.0	20.0 ± 7.0	1.6–41.3	< 0.0001	5.8 ± 2.1^*b*^	18.5 ± 7.9^*c*^	1.7–43.4	< 0.0001
Gestational age at birth (days)	276.2 ± 18.6	274.3 ± 18.1^*d*^	195–333	0.38	274.6 ± 18.7^*e*^	274.3 ± 18.1^*f*^	164–308	0.87
Gestational age at blood draw (days)	132.7 ± 26.3	120.7 ± 26.8	61–192	0.0001	134.9 ± 30.9^*g*^	119.2 ± 25.8^*h*^	47–220	< 0.0001
Maternal ethnicity				0.0041				0.61
Albanian	104 (70.8)	79 (54.9)			80 (48.5)	134 (52.6)
Serbian	33 (22.5)	39 (27.1)			56 (33.9)	75 (29.4)
Other	10 (6.8)	26 (18.1)			29 (17.6)	46 (18.0)
Smoking during pregnancy	42 (28.6)	34 (23.6)		0.34	50 (31.9)^*e*^	67 (28.9)^*f*^		0.53
Values are mean ± SD or *n *(%). Included, women still enrolled in the study who had adequate serum in storage to measure thyroid hormone and antibody levels at the 7-year follow-up. ^***a***^*n *= 254. ^***b***^*n *= 105. ^***c***^*n *= 165. ^***d***^*n *= 139. ^***e***^*n *= 157. ^***f***^*n *= 232. ^***g***^*n *= 164. ^***h***^*n *= 255.								

Among the participants, we found highly significant differences between the two towns in both FT_4_ and TPOAb (*p* < 0.0001), but not in TSH (Table 2). Women from Mitrovica, who were more highly exposed to lead (mean BPb, 20.00 vs. 5.57 μg/dL), had lower mean FT_4_ (0.91 vs. 1.03 ng/dL) and higher mean TPOAb (15.45 vs. 5.12 IU/mL), both indicative of higher risk of gestational hypothyroidism. Of the 291 women in our sample, 24 (8.25%) had FT_4_ levels < 0.7 ng/dL, the commonly used cutoff for hypothyroidism (Blatt et al. 2012), and 57 (19.59%) tested positive for TPOAb (≥ 10 IU/mL). Among those with positive TPOAb, 38 (66.67%) had slightly elevated levels (≥ 10 IU/mL and < 20 IU/mL), 13 (22.81%) had moderately elevated levels (≥ 20 IU/mL and < 100 IU/mL), and 6 (10.53%) had highly elevated levels (≥ 100 IU/mL). Most strikingly, the prevalence of elevated TPOAb (≥ 10 IU/mL) was nearly five times greater among women in Mitrovica compared with women in Pristina (32.64% vs. 6.80%) (data not shown).

**Table 2:** Comparison of BPb and thyroid measures by town among study participants.

Town	BPb (μg/dL)	FT_4_ (ng/dL)	TSH (μIU/mL)	TPOAb (IU/mL)
Pristina
*n*	147	141	142	147
Mean ± SD	5.57 ± 2.01	1.03 ± 0.16	1.46 ± 0.68	5.12 ± 6.38
Range	1.60–18.60	0.67–1.79	0.20–4.14	1.00–66.33
Mitrovica
*n*	144	138	136	144
Mean ± SD	20.00 ± 6.99	0.91 ± 0.17	1.46 ± 0.91	15.45 ± 33.08
Range	5.40–41.30	0.48–1.30	0.20–7.46	0.69–256.65
*p*-Value (ANOVA)	< 0.0001	< 0.0001	0.99	0.0002

In bivariate analyses (see Supplemental Material, Table S1), BPbs were significantly associated with town, ethnicity, maternal height, and fetal gestational age at blood draw. FT_4_ was significantly associated with ethnicity, maternal education, prepregnancy BMI, and crowded living conditions. TPOAb was significantly associated with smoking status. As expected, there was an inverse association between BMI and FT_4_. We also found that Albanians had higher mean FT_4_ than Serbians (0.99 ± 0.17 vs. 0.89 ± 0.16 ng/dL, respectively), that those with no education had higher mean FT_4_ than those with any, and that there was a positive association between FT_4_ and adults per room. These results reflect associations between height and ethnicity (*p* < 0.01), between ethnicity and education (*p* < 0.0001), and between ethnicity and adults per room (*p* < 0.001). In contrast to published findings of a protective relationship between smoking and thyroid autoimmunity (Belin et al. 2004; de Escobar et al. 2004; Effraimidis et al. 2009), in our cohort smoking was associated with a higher mean TPOAb level. TSH was not significantly associated with any of the characteristics we selected as potential covariates.

**Figure 2:**
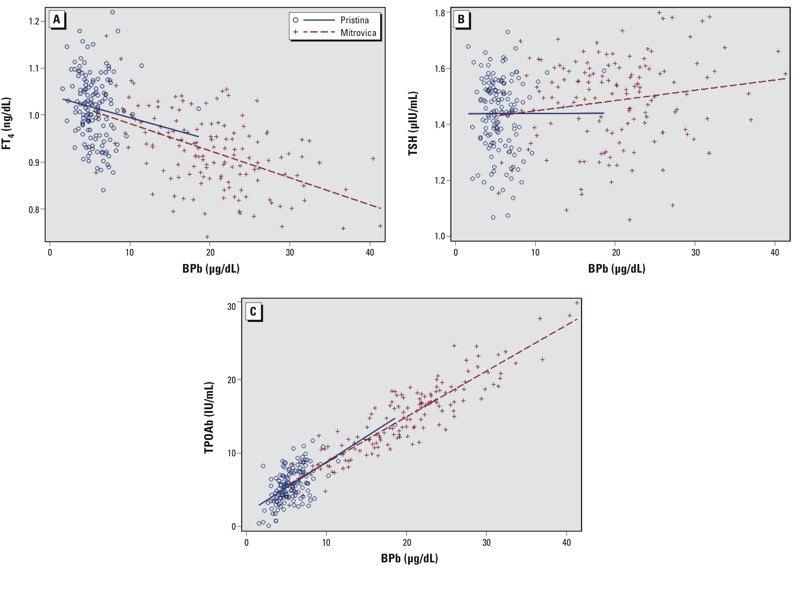
Scatter plots of measured values for each outcome according to BPb (μg/dL). (*A*) FT4 adjusted for height, ethnicity, BMI, fetal gestational age, maternal education, adults per room. (*B>*) TSH adjusted for hemoglobin, ethnicity, BMI, fetal gestational age, maternal age. (*C*) TPOAb adjusted for ethnicity, fetal gestational age, maternal age, adults per room.

Scatter plots between BPb and the three outcome variables, adjusted for potential confounders (Figure 2), suggest an inverse relationship between BPb and FT_4_ and a direct relationship between BPb and TPOAb, but no association between BPb and TSH.

BPb was negatively associated with FT_4_ and positively associated with TPOAb in both covariate adjusted and unadjusted models (*p* < 0.0001) (Table 3); no association was found between BPb and TSH. Controlling for potential confounders, for each log unit increase in BPb, FT_4_ decreased by 0.074 ng/dL (95% CI: –0.10, –0.046 ng/dL). Using logistic regression to adjust for ethnicity, fetal gestational age, maternal age, and adults per room (a proxy measure for socioeconomic status), we found the estimated odds of testing positive for TPOAb to be 2.41 times greater for every log-unit increase in mid-pregnancy BPb (95% CI: 1.53, 3.82).

**Table 3:** Unadjusted and adjusted regression coefficients (for FT_4_, ln-transformed TSH, and ln-transformed TPOAb) and odds ratios (for TPOAb ≥ 10 IU/mL vs. < 10 IU/mL) for associations with ln-transformed mid-pregnancy blood lead concentrations, Pristina and Mitrovica combined.

Outcome	Unadjusted			Adjusted		
	*R*^2^ (*n*)	β or OR (95% CI)	*p*-Value	*R*^2^ (*n*)	β or OR (95% CI)^*a*^	*p*-Value
FT_4_ (ng/dL)	0.11 (279)	–0.079 (–0.11, –0.052)	< 0.0001	0.25 (277)	–0.074 (–0.10, –0.046)	< 0.0001
ln-TSH (μIU/mL)	0.00027 (278)	–0.012 (–0.098, 0.074)	0.79	0.046 (276)	0.026 (–0.065, 0.12)	0.58
ln-TPOAb (IU/mL)	0.075 (291)	0.34 (0.20, 0.48)	< 0.0001	0.094 (291)	0.31 (0.17, 0.46)	< 0.0001
TPOAb ≥ vs. < 10 IU/mL	0.062 (291)	2.51 (1.62, 3.89)	< 0.0001	0.074 (291)	2.41 (1.53, 3.82)	0.0002
^***a***^Model covariates: FT_4_: height, ethnicity, BMI, fetal gestational age, maternal education, adults per room; TSH: hemoglobin, ethnicity, BMI, fetal gestational age, maternal age; TPOAb (continuous and dichotomous): ethnicity, fetal gestational age, maternal age, adults per room.						

## Discussion

The current study, an analysis of mid-pregnancy BPb compared with mid-pregnancy FT_4_, TSH, and TPOAb levels, yielded a highly significant negative association between BPb and FT_4_ and a highly significant positive association between BPb and TPOAb without any significant association between BPb and TSH. These results indicate that lead exposure may be a factor in reduced thyroid function, which has been suggested to increase the risk of poor obstetric outcomes (Ajmani et al. 2014; Casey et al. 2005; van den Boogaard et al. 2011) and lower IQ in children (Haddow et al. 1999; Man and Serunian 1976; Pop et al. 1995). These results suggest the plausibility of the latter of the three potential pathways by which we hypothesized that BPb might be associated with reduced thyroid function: via reduced uptake of iodine by thyroid tissue, via disruption of the release of TTR from the choroid plexus, and via the triggering of an autoimmune response to the maternal thyroid gland.

The Yugoslavia Prospective Study of Environmental Lead Exposure, Pregnancy Outcomes, and Child Development (Graziano et al. 1990) is one of several longitudinal cohort studies designed to explore the effects of long-term lead exposure on pregnant women and their offspring (Bellinger et al. 1987; Canfield et al. 2003; Dietrich et al. 1987; Ernhart et al. 1989; McMichael et al. 1988; Schnaas et al. 2006). Two towns were chosen with relatively low and high environmental lead. The mean mid-pregnancy BPb among women in Mitrovica, an industrial town with a lead smelter, refinery, and battery plant, was nearly four times higher than among women in Pristina, the capital of Kosovo (20.01 vs. 5.57 μg/dL). By contrast, according to the most comparable U.S. data available, from Phase 1 of the Third National Health and Nutrition Examination Survey (NHANES III), mean blood lead level among adults 20–49 years of age measured between 1988 and 1991 was 2.6 μg/dL (Brody et al. 1994).

Although generally considered to be a marker of recent lead exposure, BPb reflects both exogenous (environmental) and endogenous (bone, tissue) lead sources, and may also be viewed as a marker of cumulative lead exposure (Factor-Litvak et al. 1999). Indeed, because bone is remodeled during pregnancy (Hertz-Picciotto et al. 2000), BPb measured during pregnancy reflects both current and more chronic exposures. To further explore the relationship between long-term lead exposure and our outcome measures, we reran our regression models using town as the main predictor variable. We considered town to be a good proxy for long-term lead exposure because it was strongly associated with BPb in our cohort and because maternal blood samples were taken before the breakup of Yugoslavia, during a time when the residential population was relatively stable. In Mitrovica, where women were more highly exposed to lead, mean FT_4_ levels were lower (0.91 vs. 1.03 ng/dL) and TPOAb levels were higher (15.45 vs. 5.12 IU/mL) than in Pristina. Perhaps most striking, the large difference in mean TPOAb between the two towns lends credence to our hypothesis that that the relationship between long-term lead exposure and gestational thyroid dysfunction might be through the autoimmune pathway.

Although no previous studies have examined associations between lead exposure and AT or elevated TPOAb levels, studies have examined associations between other environmental exposures and the disease (e.g., Brent 2010). AT is generally acknowledged to be multifactorial, with both genetic and environmental components. Iodine has been shown to be a trigger of overt hypothyroidism in studies of patients with asymptomatic AT who were administered excessive dietary iodine (Braverman et al. 1971; Tajiri et al. 1986). Similarly, those with preexisting AT are more likely to develop hypothyroidism than those without TPOAb when given lithium (Bocchetta and Loviselli 2006). Selenium deficiency (Duntas 2010) and vitamin B_12_ deficiency (Lahner et al. 2008) have also been implicated in AT. In small observational studies, elevated TPOAb levels have been positively associated with exposure to organochlorines (Langer et al. 2008), polychlorinated biphenyls (Langer et al. 2007), and polyhalogenated biphenyls (Bahn et al. 1980). Studies using genetically predisposed mice have also shown bromine and bacterial lipopolysaccharides to be triggers of AT (Burek and Talor 2009).

Lead is known to affect the immune system, but in ways that are still not clearly understood (Dietert and Piepenbrink 2006). *In vitro* and *in vivo* studies in mice have suggested that lead initially skews T-lymphocyte response toward the Th (T helper) 2 pathway (Heo et al. 1998; McCabe and Lawrence 1991), increasing the risk of asthma and atopy, although a subsequent shift back to the Th1 pathway, observed in different mouse study, could result in a predisposition to autoimmunity (Goebel et al. 2000). In a study of mice genetically predisposed to systemic lupus erythematosus, lead exposure triggered onset of the disease (Hudson et al. 2003). Lead has also been shown to stimulate production of autoantibodies against neural proteins in both rodent models and human occupational studies (El-Fawal et al. 1999; Waterman et al. 1994).

There are several limitations to our study of lead exposure and gestational thyroid dysfunction. The sample size, though large enough to produce robust findings when data from the two towns were combined, was not large enough to support statistically significant findings when analyses were stratified by town, even though the parameter estimates were similar in the combined and stratified models (see Supplemental Material, Table S2). Although the original study sample was selected to achieve broad representation across lead exposure levels and socioeconomic status, the current study relied on the subsample for which we had mid-pregnancy thyroid measures. There is no reason to believe that such loss to follow-up would bias the biological relationships between BPb and thyroid outcome measures. Although it is possible that hormones may have degraded between the time between serum collection and analysis, we do not believe this was a major concern, because mean TSH levels are comparable to what would be expected in women during mid-pregnancy. Because thyroid binding globulin may impede the reliability of FT_4_ assays, it is preferable to use circulating total thyroxine as a measure of thyroid gland activity in pregnant women, because thyroid-binding globulin concentrations are elevated during pregnancy (Stagnaro-Green et al. 2011). Unfortunately, we did not have direct measures of total thyroxine and/or thyroid-binding globulin in our data. Finally, our data did not include mid-pregnancy urinary iodine measures, preventing us from definitively ruling out the possibility that lead causes gestational thyroid dysfunction by impairing uptake of iodine by the thyroid gland.

## Conclusions

This study contributes unique information to our understanding of lead and gestational thyroid dysfunction. Our findings suggest that long-term lead exposure increases the risk of elevated TPOAb during pregnancy, adding to the growing literature on the environmental influences on AT. Although the results of this study are limited to pregnant women, future studies might extend them to examine the effect of prenatal lead exposure on TPOAb levels in children as well as on the development of postnatal hypothyroidism among the mothers.

## Supplemental Material

(204 KB) PDF

## References

[r1] Ajmani SN, Aggarwal D, Bhatia P, Sharma M, Sarabhai V, Paul M (2014). Prevalence of overt and subclinical thyroid dysfunction among pregnant women and its effect on maternal and fetal outcome.. J Obstet Gynaecol India.

[r2] Bahn AK, Mills JL, Snyder PJ, Gann PH, Houten L, Bialik O (1980). Hypothyroidism in workers exposed to polybrominated biphenyls.. N Engl J Med.

[r3] Bath S, Steer C, Golding J, Emmett P, Rayman M (2013). Effect of inadequate iodine status in UK pregnant women on cognitive outcomes in their children: results from the Avon Longitudinal Study of Parents and Children (ALSPAC).. Lancet.

[r4] Belin R, Astor B, Powe N, Ladenson P (2004). Smoke exposure is associated with a lower prevalence of serum thyroid autoantibodies and thyrotropin concentration elevation and a higher prevalence of mild thyrotropin concentration suppression in the Third National Health and Nutrition Examination Survey (NHANES III).. J Clin Endocrinol Metab.

[r5] Bellinger D (2005). Teratogen update: lead and pregnancy.. Birth Defects Res A Clin Mol Teratol.

[r6] Bellinger D (2008). Very low lead exposures and children’s neurodevelopment.. Curr Opin Pediatr.

[r7] Bellinger D, Leviton A, Rabinowitz M, Allred E, Needleman H, Schoenbaum S (1991). Weight gain and maturity in fetuses exposed to low levels of lead.. Environ Res.

[r8] Bellinger D, Leviton A, Waternaux C, Needleman H, Rabinowitz M (1987). Longitudinal analyses of prenatal and postnatal lead exposure and early cognitive development.. N Engl J Med.

[r9] Blatt A, Nakamoto J, Kaufman H (2012). National status of testing for hypothyroidism during pregnancy and postpartum.. J Clin Endocrinol Metab.

[r10] Bledsoe M, Pinkerton L, Silver S, Deddens J, Biagini R (2011). Thyroxine and free thyroxine levels in workers occupationally exposed to inorganic lead.. Environ Health Insights.

[r11] Boas M, Forman J, Juul A, Feldt-Rasmussen U, Skakkebaek N, Hilsted L (2009a). Narrow intra-individual variation of maternal thyroid function in pregnancy based on a longitudinal study on 132 women.. Eur J Endocrinol.

[r12] Boas M, Main KM, Feldt-Rasmussen U (2009b). Environmental chemicals and thyroid function: an update.. Curr Opin Endocrinol Diabetes Obes.

[r13] Bocchetta A Loviselli A 2006 Lithium treatment and thyroid abnormalities. Clin Pract Epidemiol Ment Health 2 23; 10.1186/1745-0179-2-23 16968542 PMC1584230

[r14] Braverman LE, Ingbar SH, Vagenakis AG, Adams L, Maloof F (1971). Enhanced susceptibility to iodine myxedema in patients with Hashimoto’s disease.. J Clin Endocrinol Metab.

[r15] Brent G (2010). Environmental exposures and autoimmune thyroid disease.. Thyroid.

[r16] Brody D, Pirkle J, Kramer R, Flegal K, Matte T, Gunter E (1994). Blood lead levels in the US Population: phase 1 of the Third National Health and Nutrition Examination Survey (NHANES III, 1988 to 1991).. JAMA.

[r17] Burek CL, Talor MV (2009). Environmental triggers of autoimmune thyroiditis.. J Autoimmun.

[r18] Canfield R, Henderson C, Cory-Slechta D, Cox C, Jusko T, Lanphear B (2003). Intellectual impairment in children with blood lead concentrations below 10 μg per deciliter.. N Engl J Med.

[r19] Cantonwine D, Hu H, Sanchez B, Lamadrid-Figueroa H, Smith D, Ettinger A (2010). Critical windows of fetal lead exposure: adverse impacts on length of gestation and risk of premature delivery.. J Occup Environ Med.

[r20] Casey BM, Dashe JS, Wells CE, McIntire DD, Byrd W, Levenko KJ, Cunningham FG (2005). Subclinical hypothyroidism and pregnancy outcomes.. Obstet Gynecol.

[r21] Dietert RR, Piepenbrink MS (2006). Lead and immune function.. Crit Rev Toxicol.

[r22] Dietrich K, Krafft K, Bornschein R, Hammond P, Berger O, Succop P (1987). Low-level fetal lead exposure effect on neurobehavioral development in early infancy.. Pediatrics.

[r23] Dooyema C Neri A Lo Y-C Durant J Dargan P Swarthout T 2012 Outbreak of fatal childhood lead poisoning related to artisanal gold mining in northwestern Nigeria, 2010. Environ Health Perspect 120 601 607; 10.1289/ehp.1103965 22186192 PMC3339453

[r24] Dundar B, Oktem F, Arslan M, Delibas N, Baykal B, Arslan C (2006). The effect of long-term low-dose lead exposure on thyroid function in adolescents.. Environ Res.

[r25] Duntas LH (2010). Selenium and the thyroid: a close-knit connection.. J Clin Endocrinol Metab.

[r26] Effraimidis G, Tijssen J, Wiersinga W (2009). Discontinuation of smoking increases the risk for developing thyroid peroxidase antibodies and/or thyroglobulin antibodies: a prospective study.. J Clin Endocrinol Metab.

[r27] El-Fawal HA, Waterman SJ, Feo AD, Shamy MY (1999). Neuroimmunotoxicology: humoral assessment of neurotoxicity and autoimmune mechanisms.. Environ Health Perspect.

[r28] Ernhart C, Morrow-Tlucak M, Wolf A, Super D, Drotar D (1989). Low level lead exposure in the prenatal and early preschool periods: intelligence prior to school entry.. Neurotoxicol Teratol.

[r29] de Escobar GM, Obregon M, Rey Fd (2004). Maternal thyroid hormones early in pregnancy and fetal brain development.. Best Pract Res Clin Endocrinol Metab.

[r30] Factor-Litvak P, Graziano J, Kline J, Popovac D, Mehmeti A, Ahmedi G (1991). A prospective study of birthweight and length of gestation in a population surrounding a lead smelter in Kosovo, Yugoslavia.. Int J Epidemiol.

[r31] Factor-Litvak P, Wasserman G, Kline J, Graziano J (1999). The Yugoslavia Prospective Study of Environmental Lead Exposure.. Environ Health Perspect.

[r32] Glinoer D. (2000). Thyroid disease during pregnancy. In: Werner and Ingbar’s The Thyroid: A Fundamental and Clinical Test (Braverman L, Utiger R, eds).

[r33] Goebel C, Flohe SB, Kirchhoff K, Herder C, Kolb H (2000). Orally administered lead chloride induces bias of mucosal immunity.. Cytokine.

[r34] Gomaa A, Hu H, Bellinger D, Schwartz J, Tsaih S, Gonzalez-Cossio T (2002). Maternal bone lead as an independent risk factor for fetal neurotoxicity: a prospective study.. Pediatrics.

[r35] Gonzalez-Cossio T, Peterson K, Sanin L, Fishbein E, Palazuelos E, Aro A (1997). Decrease in birth weight in relation to maternal bone-lead burden.. Pediatrics.

[r36] Graziano J, Popovac D, Factor-Litvak P, Shrout P, Kline J, Murphy M (1990). Determinants of elevated blood lead during pregnancy in a population surrounding a lead smelter in Kosovo, Yugoslavia.. Environ Health Perspect.

[r37] Haddow J, Palomaki G, Allan W, Williams J, Knight G, Gagnon J (1999). Maternal thyroid deficiency during pregnancy and subsequent neuropsychological development of the child.. N Engl J Med.

[r38] Hartoft-Nielsen M Boas M Bliddal S Rasmussen ÅK Main K Feldt-Rasmussen U 2011 Do thyroid disrupting chemicals influence foetal development during pregnancy? J Thyroid Res; 10.4061/2011/342189 PMC3170895 21918727

[r39] Heo Y, Lee WT, Lawrence DA (1998). Differential effects of lead and cAMP on development and activities of Th1- and Th2-lymphocytes.. Toxicol Sci.

[r40] Hernandez-Avila M, Peterson K, Gonzalez-Cossio T, Sanin L, Aro A, Schnaas L (2002). Effect of maternal bone lead on length and head circumference of newborns and 1-month-old infants.. Arch Environ Health.

[r41] Hertz-Picciotto I, Schramm M, Watt-Morse M, Chantala K, Anderson J, Osterloh J (2000). Patterns and determinants of blood lead during pregnancy.. Am J Epidemiol.

[r42] Hudson CA, Cao L, Kasten-Jolly J, Kirkwood JN, Lawrence DA (2003). Susceptibility of lupus-prone NZM mouse strains to lead exacerbation of systemic lupus erythematosus symptoms.. J Toxicol Environ Health.

[r43] Lahner E, Centanni M, Agnello G, Gargano L, Vannella L, Iannoni C (2008). Occurrence and risk factors for autoimmune thyroid disease in patients with atrophic body gastritis.. Am J Med.

[r44] Lamb MR, Janevic T, Liu X, Cooper T, Kline J, Factor-Litvak P (2008). Environmental lead exposure, maternal thyroid function, and childhood growth.. Environ Res.

[r45] Langer P, Kocan A, Tajtakova M, Koska J, Radikova Z, Ksinantova L (2008). Increased thyroid volume, prevalence of thyroid antibodies and impaired fasting glucose in young adults from organochlorine cocktail polluted area: outcome of transgenerational transmission?. Chemosphere.

[r46] Langer P, Kocan A, Tajtakova M, Petrik J, Chovancova J, Drobna B (2007). Fish from industrially polluted freshwater as the main source of organochlorinated pollutants and increased frequency of thyroid disorders and dysglycemia.. Chemosphere.

[r47] Lanphear B Hornung R Khoury J Yolton K Baghurst P Bellinger D 2005 Low-level environmental lead exposure and children’s intellectual function: an international pooled analysis. Environ Health Perspect 113 894 899; 10.1289/ehp.7688 16002379 PMC1257652

[r48] Lazarus J, Bestwick J, Channon S, Paradice R, Maina A, Rees R (2012). Antenatal thyroid screening and childhood cognitive function.. N Engl J Med.

[r49] Man E, Serunian S (1976). Thyroid function in human pregnancy. IX. Development or retardation of 7-year-old progeny of hypothyroxinemic women.. Am J Obstet Gynecol.

[r50] Mannisto T, Surcel H, Bloigu A, Ruokonen A, Hartikainen A, Jarvelin M (2007). The effect of freezing, thawing, and short- and long-term storage on serum thyrotropin, thyroid hormones, and thyroid autoantibodies: implications for analyzing samples stored in serum banks.. Clin Chem.

[r51] Mbah A, Ejim E, Onodugo O, Ezugwu F, Eze M, Nkwo P (2011). Two logistic models for the prediction of hypothyroidism in pregnancy.. BMC Res Notes.

[r52] McCabe MJ, Lawrence DA (1991). Lead, a major environmental pollutant, is immunomodulatory by its differential effects on CD4^+^ T cell subsets.. Toxicol Appl Pharmacol.

[r53] McMichael A, Baghurst P, Wigg N, Vimpani G, Robertson E, Roberts R (1988). Port Pirie Cohort Study: environmental exposure to lead and children’s abilities at the age of four years.. N Engl J Med.

[r54] Needleman H, Landrigan P (1991). The health effects of low level exposure to lead.. Annu Rev Public Health.

[r55] Opler MGA Brown AS Graziano J Desai M Zheng W Schaefer C 2004 Prenatal lead exposure, delta-aminolevulinic acid, and schizophrenia. Environ Health Perspect 112 548 552; 10.1289/ehp.6777 15064159 PMC1241919

[r56] Opler MGA Buka SL Groeger J McKeague I Wei C Factor-Litvak P 2008 Prenatal exposure to lead, δ-amniolevulinic acid, and schizophrenia: further evidence. Environ Health Perspect 116 1586 1590; 10.1289/ehp.10464 19057716 PMC2592283

[r57] Pearce EN, Braverman LE (2009). Environmental pollutants and the thyroid.. Best Pract Res Clin Endocrinol Metab.

[r58] Pop V, deVries E, vanBaar A, Waelkens J, deRooy H, Horsten M (1995). Maternal thyroid peroxidase antibodies during pregnancy: a marker of impaired child development?. J Clin Endocrinol Metab.

[r59] Popovac D, Graziano J, Seaman C, Kaul B, Colakovic B, Popovac R (1982). Elevated blood lead in a population near a lead smelter in Kosovo, Yugoslavia.. Arch Environ Health.

[r60] Sandstead H, Stant E, Brill A, Arias L, Terry R (1969). Lead intoxication and the thyroid.. Arch Intern Med.

[r61] Schnaas L Rothenberg SJ Flores F Martinez S Hernandez C Osorio E 2006 Reduced intellectual development in children with prenatal lead exposure. Environ Health Perspect 114 791 797; 10.1289/ehp.8552 16675439 PMC1459938

[r62] Slingerland D (1955). The influence of various factors on the uptake of iodine by the thyroid.. J Clin Endocrinol Metab.

[r63] Stagnaro-Green A, Abalovich M, Alexander E, Azizi F, Mestman J, Negro R (2011). Guidelines of the American Thyroid Association for the diagnosis and management of thyroid disease during pregnancy and postpartum.. Thyroid.

[r64] Stagnaro-Green A, Pearce EN (2013). Iodine and pregnancy: a call to action.. Lancet.

[r65] Tajiri J, Higashi K, Morita M, Umeda T, Sato T (1986). Studies of hypothyroidism in patients with high iodine intake.. J Clin Endocrinol Metab.

[r66] Tuppurainen M, Wagar G, Kurppa K, Sakari W, Wambugu A, Froseth B (1988). Thyroid function as assessed by routine laboratory tests of workers with long-term lead exposure.. Scand J Work Environ Health.

[r67] van den Boogaard E, Vissenberg R, Land JA, van Wely M, van der Post JA, Goddijn M (2011). Significance of (sub)clinical thyroid dysfunction and thyroid autoimmunity before conception and in early pregnancy: a systematic review.. Hum Reprod Update.

[r68] Wasserman G, Liu X, Popovac D, Factor-Litvak P, Kline J, Waternaux C (2000). The Yugoslavia Prospective Lead Study: contributions of prenatal and postnatal lead exposure to early intelligence.. Neurotoxicol Teratol.

[r69] Wasserman G, Staghezza-Jaramillo B, Shrout P, Popovac D, Graziano J (1998). The effect of lead exposure on behavior problems in preschool children.. Am J Public Health.

[r70] Waterman SJ, El-Fawal HA, Snyder CA (1994). Lead alters the immunogenicity of two neural proteins: a potential mechanism for the progression of lead-induced neurotoxicity.. Environ Health Perspect.

[r71] Zheng W, Lu Y, Lu G, Zhao Q, Cheung O, Blaner W (2001). Transthyretin, thyroxine, and retinol-binding protein in human cerebrospinal fluid: effect of lead exposure.. Toxicol Sci.

[r72] Zheng W, Shen H, Blaner W, Zhao Q, Ren X, Graziano J (1996). Chronic lead exposure alters transthyretin concentration in rat cerebrospinal fluid: the role of the choroid plexus.. Toxicol Appl Pharmacol.

