# Supplementary material for: Blood Lead Concentration and Thyroid Function during Pregnancy: Results from the Yugoslavia Prospective Study of Environmental Lead Exposure
Source: Environ Health Perspect. 2014 May 27;122(10):1134–40. doi: 10.1289/ehp.1307669 (PMC4181923; doi:10.1289/ehp.1307669)
Supplement: (204 KB) PDF [file ehp.1307669.s001.508.pdf]

Environmental Health Perspectives doi:10.1289/ehp.1307669

Supplemental Material

Blood Lead Concentration and Thyroid Function during Pregnancy: Results from the Yugoslavia Prospective Study of Environmental Lead Exposure

Linda G. Kahn, Xinhua Liu, Biljana Rajovic, Dusan Popovac, Sharon Oberfield, Joseph H. Graziano, and Pam Factor-Litvak

Table S1. Bivariate associations between participant characteristics and mid-pregnancy thyroid function parameters [BPb (µg/dL), FT4 (ng/dL), TSH (µIU/mL), and TPO Ab (IU/mL)] among study participants.

Characteristic	BPb n	BPb Mean ± SD	p-value[a]	FT4 n	FT4 Mean ± SD	p-value[a]	TSH n	TSH Mean ± SD	p-value[a]	TPO Ab n	TPO Ab Mean ± SD	p-value[a]
**Town**			<0.0001			<0.0001			0.99			0.0002
Mitrovica	144	20.00 ± 6.99		138	0.91 ± 0.17		136	1.46 ± 0.91		144	15.45 ± 33.08	
Pristina	147	5.57 ± 2.01		141	1.03 ± 0.16		142	1.46 ± 0.68		147	5.12 ± 6.38	
**Ethnicity**	291		0.0019	279		<0.0001	278		0.13	291		0.46
Albanian	184	11.48 ± 8.41		178	0.99 ± 0.17		177	1.52 ± 0.85		184	9.06 ± 22.74	
Serbian	71	13.86 ± 8.97		67	0.89 ± 0.16		68	1.38 ± 0.75		71	13.29 ± 29.51	
Other	36	16.77 ± 9.55		34	1.02 ± 0.19		33	1.26 ± 0.53		36	10.24 ± 19.76	
**Maternal education**	291		0.64	279		0.030	278		0.34	291		0.62
None	16	13.51 ± 11.09		16	1.08 ± 0.25		16	1.15 ± 0.62		16	12.24 ± 30.79	
1-8 years	113	11.88 ± 8.26		109	0.98 ± 0.18		108	1.53 ± 0.75		113	8.11 ± 13.44	
9-12 years	143	13.27 ± 9.12		135	0.95 ± 0.17		135	1.44 ± 0.87		143	11.93 ± 30.73	
≥ 13 years	19	12.79 ± 8.44		19	0.97 ± 0.12		19	1.43 ± 0.63		19	8.44 ± 5.72	
**Prior live births**	291		0.075	279		0.46	278		0.53	291		0.28
0	88	14.39 ± 8.79		86	0.97 ± 0.16		83	1.54 ± 1.00		88	13.66 ± 32.67	
1-2	143	11.66 ± 8.57		139	0.96 ± 0.16		141	1.42 ± 0.69		143	8.50 ± 19.99	
3 or more	60	12.75 ± 9.34		54	1.00 ± 0.23		54	1.44 ± 0.73		60	9.35 ± 17.95	
**Pre-pregnancy BMI**	289		0.47	277		0.0081	276		0.84	289		0.84
Normal (< 25 kg/m²)	165	12.34 ± 8.96		156	1.00 ± 0.18		157	1.48 ± 0.78		165	9.54 ± 24.39	
Overweight (25-29.9 kg/m²)	94	12.96 ± 8.73		92	0.94 ± 0.16		90	1.42 ± 0.86		94	11.13 ± 26.67	
Obese (≥ 30 kg/m²)	30	14.48 ± 8.75		29	0.92 ± 0.16		29	1.43 ± 0.75		30	11.60 ± 14.54	
**Smoking status**	291		0.84	279		0.89	278		0.18	291		0.037
Nonsmoker	215	12.65 ± 8.75		208	0.97 ± 0.18		206	1.42 ± 0.66		215	8.47 ± 13.68	
Current smoker	76	12.89 ± 9.19		71	0.97 ± 0.16		72	1.57 ± 1.10		76	15.22 ± 41.24	
**Hemoglobin**	290		0.12	278		0.80	278		0.19	290		0.71
Sufficient (≥ 10.5 g/dL)	275	12.88 ± 8.88		264	0.97 ± 0.17		265	1.44 ± 0.80		275	10.34 ± 24.87	
Deficient (< 10.5 g/dL)	15	9.21 ± 7.98		14	0.98 ± 0.21		13	1.74 ± 0.67		15	7.90 ± 6.26	
**Crowded living conditions**	291		0.18	279		0.027	278		0.39	291		0.97
No (≤ 1.33 adults/room)	142	13.42 ± 8.88		137	0.95 ± 0.18		136	1.41 ± 0.67		142	10.18 ± 21.98	
Yes (> 1.33 adults/room)	149	12.04 ± 8.80		142	0.99 ± 0.17		142	1.50 ± 0.90		149	10.29 ± 26.26	
**Maternal age (years)**	291		0.63	279		0.90	278		0.32	291		0.72
< 20	16	14.13 ± 7.99		16	0.97 ± 0.18		15	1.51 ± 0.61		16	5.08 ± 3.58	
20-24	107	11.86 ± 8.62		103	0.97 ± 0.18		101	1.56 ± 0.95		107	10.92 ± 28.94	
25-29	97	13.09 ± 9.02		92	0.98 ± 0.18		94	1.35 ± 0.63		97	9.14 ± 16.85	
≥ 30	71	13.15 ± 9.21		68	0.96 ± 0.17		68	1.43 ± 0.78		71	11.86 ± 27.55	
**Gestational age at blood draw (days)**	291		0.022	279		0.25	278		0.71	291		0.32
< 106	70	15.20 ± 9.29		66	1.01 ± 0.17		66	1.36 ± 0.83		70	13.98 ± 30.91	
106-125	66	12.93 ± 9.22		62	0.97 ± 0.17		64	1.52 ± 0.77		66	9.48 ± 17.00	
126-145	79	12.20 ± 8.78		76	0.95 ± 0.17		74	1.49 ± 0.69		79	11.05 ± 32.20	
≥ 146	76	10.76 ± 7.72		75	0.96 ± 0.18		74	1.46 ± 0.90		76	6.61 ± 5.80	
**Maternal height (cm)**	289		<0.0001	277		0.058	276		0.058	289		0.33
< 155	34	16.26 ± 9.67		29	0.96 ± 0.16		30	1.31 ± 0.56		34	9.48 ± 17.53	
155-159	72	16.25 ± 9.50		69	0.93 ± 0.18		71	1.67 ± 1.07		72	13.14 ± 30.83	
160-164	121	10.30 ± 7.62		119	1.00 ± 0.18		117	1.38 ± 0.67		121	7.39 ± 12.49	
≥ 165	64	11.39 ± 8.08		62	0.97 ± 0.16		60	1.43 ± 0.70		64	12.74 ± 33.76	
**Pre-pregnancy weight (kg)**	289		0.77	277		0.076	276		0.47	289		0.66
< 55	48	12.77 ± 9.77		43	1.03 ± 0.18		45	1.41 ± 0.54		48	9.30 ± 17.54	
55-64	108	13.22 ± 9.03		103	0.98 ± 0.18		104	1.54 ± 0.92		108	8.85 ± 22.76	
65-74	86	11.89 ± 8.66		86	0.95 ± 0.17		84	1.44 ± 0.82		86	13.00 ± 32.83	
≥ 74	49	12.97 ± 7.95		47	0.95 ± 0.16		45	1.33 ± 0.63		49	9.34 ± 12.68	

[a] p-values determined by analysis of variance.

Table S2. Unadjusted and adjusted regression coefficients (for free T4, ln-transformed TSH, and ln-transformed TPO Ab) and odds ratios (for TPO Ab ≥ 10 IU/mL versus < 10 IU/mL) for associations with ln-transformed mid-pregnancy blood lead concentrations, Pristina and Mitrovica.

Outcome	Unadjusted R^2 (n)	Unadjusted β or OR (95% CI)	p-value	Adjusted R^2 (n)	Adjusted β or OR (95% CI)[a]	p-value
**Pristina**						
Free T4 (ng/dL)	0.0022 (141)	-0.023 (-0.11, 0.059)	0.58	0.23 (139)	-0.033 (-0.11, 0.046)	0.41
Ln-TSH (μIU/mL)	0.0024 (142)	0.041 (-0.10, 0.18)	0.57	0.069 (140)	0.054 (-0.91, 0.20)	0.46
Ln-TPO Ab (IU/mL)	0.015 (147)	-0.22 (-0.53, 0.092)	0.17	0.070 (147)	-0.22 (-0.54, 0.087)	0.16
TPO Ab ≥ vs. < 10 IU/mL	0.00010 (147)	1.1 (0.17, 7.28)	0.92	0.061 (147)	1.29 (0.16, 10.52)	0.81
**Mitrovica**						
Free T4 (ng/dL)	0.019 (138)	-0.060 (-0.13, 0.014)	0.11	0.17 (138)	-0.053 (-0.13, 0.021)	0.16
Ln-TSH (μIU/mL)	0.0013 (136)	0.030 (-0.11, 0.17)	0.68	0.12 (136)	0.062 (-0.080, 0.21)	0.39
Ln-TPO Ab (IU/mL)	0.00096 (144)	-0.083 (-0.53, 0.36)	0.71	0.037 (144)	-0.12 (-0.57, 0.33)	0.59
TPO Ab ≥ vs. < 10 IU/mL	0.012 (144)	0.54 (0.22, 1.34)	0.19	0.044 (144)	0.49 (0.19, 1.26)	0.14

[a]Model covariates: Free T4 = height, ethnicity, BMI, fetal gestational age, maternal education, adults per room; TSH = hemoglobin, ethnicity, BMI, fetal gestational age, maternal age; TPO Ab (continuous and dichotomous) = ethnicity, fetal gestational age, maternal age, adults per room
